# Neutropenia in patients treated with clozapine during COVID-19 infection

**DOI:** 10.1192/j.eurpsy.2021.1748

**Published:** 2021-08-13

**Authors:** J. Marti Bonany, M. Pérez Carre, J.R. Fortuny Olive, C. Macias Castellví, E. Carrió Diez, R. Sánchez González, M. Vallve Elias, G.A. Mateu Codina, M.I. Martínez Casamitjana

**Affiliations:** Institut De Neuropsiquiatria I Addiccions, Parc de Salut Mar, Santa Coloma de Gramenet, Spain

**Keywords:** clozapine, neutropenia, COVID-19

## Abstract

**Introduction:**

Clozapine is the most effective antipsychotic for treatment resistant schizophrenia but adverse reactions to clozapine include neutropenia. Patients with COVID-19 infection frequently experience lymphopenia, but not neutropenia.The impact of clozapine treatment in the presence of COVID-19 is unknown

**Objectives:**

Show 2 cases of neutropenia in patients treated with long-term clozapine during COVID-19 infection.

**Methods:**

Subjects: 48 admitted patients to a long-stay psychiatric unit. COVID-19 infection confirmed by positive nasopharyngeal swab for viral ribonucleic acid of SARS-CoV-2. Hematological controls between March and April 2020.

**Results:**

16 patients (33%) treated with clozapine.18 patients (37’5%) had COVID-19 infection, of which 5 (10’4%) were treated with clozapine; 2 presented neutropenia. 1- 56-year-old woman diagnosed with schizophrenia on clozapine since 2009. Begins to have a dry cough and fever with positive COVID-19 swab (day 0). Slight leukopenia without neutropenia was observed on day 1. On day 7, neutropenia was observed with an absolute neutrophil count (ANC) of 1100. We decided to suspend clozapine and to initiate daily hematological controls. The ANC on day 8 was 970. Over the next few days the ANC will progressively improve until neutropenia resolved (day 22). 2- 55-year-old woman who required a transfer to a general hospital because of respiratory complications from COVID-19. She presented significant leukopenia (1’01x 10^3/uL) and neutropenia (ANC 100). Clozapine was not withdrawn. She was treated with granulocyte colony-stimulating factor.

**Conclusions:**

An urgent full blood count will be required to exclude neutropenia with appropriate action. Further research will be needed to clarify the possible relationship between COVID-19, clozapine and neutropenia.

**Disclosure:**

No significant relationships.

